# List of Original Papers in the British Medical Journals for the Quarter Ending the 20th Dec. 1842

**Published:** 1843-01

**Authors:** 


### ORIGINAL PAPERS IN THE BRITISH MEDICAL JOURNALS,

For the Quarter ending 20th Dec. 1842.

### ANATOMY, PHYSIOLOGY, AND MORBID ANATOMY.

| | | |
|---|---|---|
| Dr. Laycock—On a general Law of vital Periodicity | *Lancet,* | Oct. 22. |
| On Periodicity | *Ibid.* | Dec. 17. |
| Dr. Collier—On Intra-combustion | *Ibid.* | Oct. 22. |
| Dr. Barry—On the Production of Muscle | *Ibid.* | Oct. 29. |
| Mr. Ancell—On the Doctrines of Liebig | *Ibid.* | Nov. 12. |
| Dr. Watts—On Coagulation of the Blood | *Ibid.* | Nov. 12. |
| Mr. Shaw—On the Circulation of the Liver | *Med. Gaz.* | Sept. 30. |
| Mr. Heron—Absorption of the Stomach of a Gull | *Dub. Press,* | Oct. 12. |
| Mr. Adams—Case of Somnambulism and Clairvoyance | *Med. Times,* | Oct. 15. |
| Mr. Prankerd—A Man with three Testicles | *Prov. Times,* | Nov. 19. |
| Dr. Macartney—On the Brain of the Chimpanzee | *Dublin Press,* | Nov. 16. |
| Dr. Anderson—Anatomy of Cauliflower Excrescence | *Edin. Med. Journ.* | Oct. |
| Mr. Adams—Case of Clairvoyance | *Med. Times,* | Oct. 15. |
| Mr. Kesteven—Influence of Mothers' Imagination | *Med. Times,* | Nov. 19. |
| Mr. Day—Tumour of the Dura Mater | *Prov. Journ.* | Dec. 3. |
| Dr. Hocken—On the Action of the Muscles of the Eye | *Edin. Journ.* | Oct. 1. |
| Mr. Spence—Anatomy of the Par Vagum | *Ibid.* | Oct. 1. |
| Mr. Ewart—On the Skin and Mucous Membrane | *Ibid.* | Oct. 1. |

### PATHOLOGY, PRACTICAL MEDICINE, AND THERAPEUTICS.

| | | |
|---|---|---|
| Mr. Orwin—On Epilepsy from protracted Lactation | *Prov. Jour.* | Oct. 15. |
| Mr. West—On the Diagnosis of Cholera | *Ibid.* | Oct. 15. |
| Dr. Beverly—Case of Hemiplegia | *Ibid.* | Oct. 22. |
| Dr. Knight—Effects of disorder of Colon | *Ibid.* | Oct. 22. |
| Dr. Hastings—On the Cold Water Cure | *Ibid.* | Oct. 22. |
| Mr. Barrett—On the Diagnosis of Cholera | *Ibid.* | Oct. 29. |
| Mr. Sadler—On the Diagnosis of Cholera | *Ibid.* | Nov. 5. |
| M. Combette—Diabetes cured by Iodide of Iron | *Ibid.* | Nov. 5. |
| Mr. Edwards—Case of Spasmodic Cholera | *Ibid.* | Nov. 12. |
| Mr. West—On the Diagnosis of Cholera | *Ibid.* | Nov. 12. |
| Dr. Kane—Medical Cases | *Dublin Press,* | Sept. 28. |
| Mr. Catty—On the waters of Ems | *Ibid.* | Oct. 12. |
| Mr. Donovan—On the Effects of a New Remedy | *Dublin Journal,* | Nov, 1. |
| Dr. Hastings—Illustrations of the Water Cure | *Prov. Jour.* | Nov. 19. |
| Mr. Macintire—Case of Hydrophobia | *Dublin Press,* | Nov. 16. |
| Dr. Johnson—On Epidemie Erysipelas | *Medical Times,* | Oct. 29. |
| Dr. Clay—On Secale Cornutum | *Ibid.* | Oct. 29. |
| Mr. Gore—On the Secale Cornutum | *Ibid.* | Nov. 5. |
| Mr. Johnson—Case of Tetanus treated by Opium | *Ibid.* | Nov. 5. |
| Mr. Greetwood—On Secale Cornutum | *Ibid.* | Nov. 12. |
| Mr. Lewis—On the Treatment of Apoplexy | *Medical Gazette,* | Nov. 25. |
| Mr. Allison—On the Diagnosis of Cholera | *Prov. Jonr.* | Nov. 26. |
| Mr. Toogood—Brain Affection after Scarlatina | *Ibid.* | Nov. 26. |
| Mr. Cheyne—Cure of Hay Fever | *Medical Gazette,* | Dec. 2. |
| Mr. Roberts—On the Health of Soldiers at Sea | *Ibid.* | Dec. 2. |
| Dr. Kinnis—On Tubercular Leprosy | *Edin. Jour.* | Oct. 1. |
| Mr. Prior—On Indian Dysentery | *Ibid.* | Oct. 1. |
| Dr. Bennett—On Inflammation of the Nervous Centres | *Ibid.* | Oct. 1. |
| Dr. Strong—On Sulphate of Zinc in Bowel Complaints | *Ibid.* | Oct. 1. |
| Mr. Worthington—Case of Cerebral Affection | *Prov. Jour.* | Dec. 10. |
| Mr. Jameson—On Swelled Leg after Fever | *Ibid.* | Dec. 10. |
| Dr. Fife—On the Diagnosis of Cholera | *Ibid.* | Dec. 10. |
| Mr. Perry—Case of Puerperal Mania | *Lancet,* | Dec. 10. |
| Mr. Hitchman—On the Effect of mental Impressions | *Med. Gaz.* | Dec. 10. |

| | | |
|---|---|---|
| Dr. SILVESTER—Death from Hydropathy | *Med. Gaz.,* | Dec. 16. |
| Dr. MACLACHLAN—On a variety of Diarrhœa | *Ibid.* | Dec. 16. |
| Dr. WATTS—On Paralysis from Derangement of the Ganglionic System, | *Lancet,* | Dec. 17. |
| Mr. BENSON—On preventing Lead Colic | *Ibid.* | Dec. 17. |
| Mr. BEDDOW—On Scarlatina | *Ibid.* | Dec. 17. |
| Dr. EPPS—Case of Giddiness in a Child | *Lancet,* | Oct. 8. |
| Mr. DAVEY—Cholera from disease of the Solar Plexus | *Ibid.* | Oct. 22. |
| Dr. HARE—On Milk as the cause of Tubercles | *Ibid.* | Oct. 29. |
| Sir G. LEFEVRE—On the frequency of Pulmonary Disease | *Ibid.* | Nov. 5. |
| Dr. ALDIS—Cases of Fits | *Ibid.* | Nov. 5. |
| Messrs. HORNE, &c.—On the use of Catechu in Sore Nipples | *Ibid.* | Nov. 12. |
| Dr. DICK—On the Cold-water Cure | *Ibid.* | Nov. 12. |
| Mr. MORE—On Diseases of the Heart and Arteries | *Med. Gaz.* | Sep. 30. |
| Mr. DOUGLAS—Case of Blue Disease | *Ibid.* | Sep. 30. |
| Dr. AYRES—Case of Asiatic Cholera | *Ibid.* | Sep. 30. |
| Mr. CHALMERS—Remarkable case of Constipation | *Ibid.* | Sep. 30. |
| Mr. FLUDER—Cases of Acute Hydrocephalus | *Ibid.* | Sep. 30. |
| Mr. BELLINGHAM—Case of Aneurism of the External Iliac | *Ibid.* | Sep. 30. |
| Mr. STAINTHORPE—Case of Purpura Hæmorrhagica | *Ibid.* | Oct. 14. |
| Mr. BARTRUM—Case of Albuminous Urine | *Ibid.* | Oct. 14. |
| Mr. COPEMAN—On Diseases of the Brain | *Ibid.* | Oct. 21. |
| Mr. ROBBS—Case of Ascites | *Ibid.* | Nov. 4. |
| Dr. MAYO—On Nervous Apoplexy | *Ibid.* | Nov. 11. |
| Dr. BUDD—Case of Enlarged Bronchial Glands | *Ibid.* | Nov. 11. |
| Mr. ADDISON—On the nature of Tubercles | *Ibid.* | Nov. 11. |
| Mr. SALTER—On the Diagnosis of Cholera | *Prov. Jour.* | Oct. 8. |
| Mr. EVANS—Case of Anomalous Hysteria | *Ibid.* | Oct. 8. |
| ANON.—Case of Abscess of the Lungs | *Ibid.* | Oct. 8. |
| Dr. BROWNE—Case of Abscess of the Brain | *Ibid.* | Oct. 8. |
| Dr. HOCKEN—Case of Fatal Pharyngeal Abscess | *Ibid.* | Oct. 8. |
| Dr. WATERS—On Tobacco Injections in Enteritis | *Ibid.* | Oct. 15. |
| Dr. O'BEIRNE—On the Nature and Treatment of Dropsy | *Dub. Journ.* | Nov. 1. |

### SURGERY, INCLUDING DISEASES OF THE EYE AND EAR.

| | | |
|---|---|---|
| Mr. TOOGOOD—On Mild Mercurial Friction | *Prov. Jour.* | Oct. 15. |
| Mr. CRABB—Hemorrhage from extraction of a Tooth | *Ibid.* | Oct. 22. |
| Mr. ELLIOTT—Case of Calculus | *Ibid.* | Oct. 29. |
| Mr. ALLISON—Excision of the Cervix uteri | *Ibid.* | Oct. 29. |
| Mr. WORTHINGTON—Case of Ununited Fracture | *Ibid.* | Oct. 29. |
| Mr. GRIFFITH—Tartar emetic affecting the Scrotum | *Ibid.* | Nov. 12. |
| Mr. CLEAVE—Treatment of Ununited Fracture | *Ibid.* | Nov. 12. |
| Dr. KANE—Surgical Cases | *Dublin Press,* | Sep. 28. |
| Mr. KIRBY—Infantile Luxation of the Femur | *Ibid.* | Oct. 26. |
| Dr. KINGSLEY—Case of Blue Foot | *Ibid.* | Oct. 26. |
| Mr. CLAY—Cases of Extirpation of the Ovarium | *Med. Times,* | Oct. 15. |
| Dr. LIDDLE—On Asphyxia in a Diver | *Med. Chir. Rev.* | Oct. 1. |
| Mr. BELL—Cases of Strangulated Hernia | *Edin. Monthly Jour.* | Nov. 1. |
| Mr. SYME—Surgical Cases and Observations | *Ibid.* | Nov. 1. |
| Dr. HAMILTON—Case of Femoral Aneurism | *Dub. Jour.* | Nov. 1. |
| Mr. TANNER—Cause of Cauliflower Excrescences | *Prov. Jour.* | Nov. 19. |
| Dr. HOCKEN—On Strumous Conjunctivites | *Lancet,* | Nov. 19. |
| Dr. CLAY—Cases of Extirpation of the Ovary | *Med. Times,* | Oct. 15. |
| Mr. THOMAS—Escape of Calculi through the Abdomen | *Ibid.* | Nov. 12. |
| Mr. SMITH—Case of Gun-shot Wound | *Ibid.* | Nov. 26. |
| Mr. DOMVILLE—Cases of Rupture of ovarian Sac | *Med. Gaz.* | Nov. 25. |
| Mr. WOOD—Mode of preventing Sore Nipples | *Lancet,* | Nov. 26. |
| Mr. WORMALD—On the Treatment of Ophthalmy | *Ibid.* | Nov. 26. |
| Mr. PITT—Tartar Emetic Affecting the Scrotum | *Prov. Jour.* | Nov. 26. |
| Mr. SYME—On the Operation of Lithotomy | *Edin. Month. Jour.* | Dec. 1. |
| Mr. PARKER—On Unusual Forms of Dislocation | *Ibid.* | Dec. 1. |
| Mr. ADAMS—On Impaired Vision | *Med. Gaz.* | Dec. 16. |
| Dr. KING—Rare case of Hernia | *Ibid.* | Dec. 16. |
| Mr. ADAMS—Dislocation of the Knee-joint | *Ibid.* | Dec. 16. |
| Dr. WALLACE—On Myopia | *Ibid.* | Dec. 16. |

| | |
|---|---|
| Mr. Gray—On Strangulated Hernia | *Lancet*, Dec. 17. |
| Mr. Eagle—On Excision of the Spleen as a Curative Means | *Ibid.* Oct. 8. |
| Mr. Keir—Case of diseased Vision | *Ibid.* Oct. 8. |
| Mr. James—Case of Retention of Urine | *Lancet*, Oct. 8, *Med. Gaz.* 7. |
| Dr. Hutchinson—On Idiopathic Gangrene | *Lancet*, Oct. 15. |
| Mr. Gay—On Amputation below the Knee | *Ibid.* Oct. 22. |
| Mr. Lizars—Case of Sarcoma of the Mamma | *Ibid.* Oct. 22. |
| Mr. Reeves—Treatment of Erysipelas by Iodine | *Ibid.* Oct 22. |
| Dr. Laycock—On a General Law of Vital Periodicity | *Ibid.* Oct. 22. |
| Mr. Denton—Mode of clearing a Clogged Catheter | *Ibid.* Oct. 29. |
| Dr. Turnbull—New treatment of Disease of the Eye, &c. | *Ibid.* Oct. 29. |
| Mr. Moore—Case of fatal blow on the Lung | *Ibid.* Nov. 5. |
| Mr. Crisp—On Idiopathic Gangrene | *Ibid.* Nov. 5. |
| Mr. Macann—A Man with three Testicles | *Ibid.* Nov. 5. |
| Mr. Lampard—On clogged Catheters | *Ibid.* Nov. 12. |
| Mr. Smee—Treatment of Syphilis by Antimony | *Med. Gaz.* Oct. 7. |
| Mr. May—Surgical Cases | *Ibid.* Oct. 7. |
| Mr. Grantham—Treatment of Fractures | *Ibid.* Oct. 14. |
| Dr. Laurie—On various forms of Aneurism | *Ibid.* Oct. 21. |
| Mr. Stanger—Case of Tracheotomy | *Ibid.* Oct. 21. |
| Mr. Thornton—Case of Deafness | *Ibid.* Oct. 28. |
| Mr. Knox—Abrasion and Atrophy of Cartilages | *Ibid.* Nov. 4. |
| Dr. Turnbull—On bisulphuret of Carbon, in Diseases of the Eye | *Ibid.* Nov. 4. |
| Mr. Sutton—Case of Entero-epiplocele | *Ibid.* Nov. 11. |
| Mr. Allison—Case of Abscess of the Prostate | *Prov. Journ.* Oct. 1. |
| Mr. Storrs—On the operation for Ovarian Dropsy | *Ibid.* Oct. 1. |
| Mr. Cooper—Melanoid Tumour of the Eye | *Med. Gaz.* Dec. 2. |
| Mr. Shortridge—Case of Empyema | *Ed. Journ.* Oct. 1. |
| Mr. Rhind—A new remedy for Burns | *Ibid.* Oct. 1. |
| Mr. Slater—On Tartar Emetic in Joint Disease | *Prov. Journ.* Dec. 10. |
| Mr. Estlin—On pretended Cases of Cataract | *Ibid.* Dec. 10. |
| Mr. Soden—Dislocated tendon of the Biceps | *Med. Gaz.* Dec. 9. |
| Dr. Furnivall—On Iodine in Conjunctivitis | *Lancet*, Dec. 10. |

### MIDWIFERY, AND DISEASES OF WOMEN AND CHILDREN.

| | |
|---|---|
| Mr. Hunter—On the duration of Labour | *Lancet*, Nov. 5. |
| Mr. Iliff—On protracted Suckling | *Ibid.* Nov. 12. |
| Mr. Bell—Case of Bony Placenta | *Med. Gaz.* Oct. 21. |
| Mr. Coward—Case of Laceration of the Perineum | *Ibid.* Oct. 28. |
| Mr. Snow—On Uterine Hemorrhage | *Ibid.* Nov. 11. |
| Dr. Laycock—Influence of Lactation on Impregnation | *Dub. Press,* Oct. 26. |
| Dr. Smith—On the Obstetric Perforator | *Edin. Month. Journ.* Nov. 1. |
| Dr. Doherty—On Inflammation of the Uterine Appendages | *Dub. Journ.* Nov. 1. |
| Dr. Hennen—On the Mortality of Children | *Prov. Journ.* Nov. 19. |
| Mr. Haden—On Leeching the Os Uteri | *Lancet,* Nov. 19. |
| Dr. Reid—On the vessels of the Placenta | *Med. Gaz.* Nov. 18. |
| Mr. Turner—On the length of the Funis | *Lancet,* Nov. 26. |
| Mr. Turner—On Births by Paraplegic Women | *Ibid.* Nov. 26. |
| Mr. Craig—On Laudanum and Spirit after Parturition | *Ibid.* Nov. 26. |
| Dr. Griffifth—Character of the Urine in Pregnancy | *Edin. Month. Jour.* Dec. 1. |
| Dr. M'Cormac—On the period of Puberty in Negro Women | *Med. Gaz.* Dec. 2. |
| Dr. Paterson—On the Corpora Lutea | *Ibid.* Dec. 9. |

### MATERIA MEDICA, PHARMACY, AND CHEMISTRY.

| | |
|---|---|
| Dr. Burton—On Equivalent Doses of different Med. Preparations | *Med. Gaz.* Oct. 7. |
| Dr. Griffith—On Albuminous Urine, &c. | *Ibid.* Oct. 21. |
| Dr. Bird—On Animal Matter in the Urine | *Edin. Month. Journ.* Nov. 1. |
| Dr. Pereira—On the Fruits of Hemlock, &c. | *Pharm. Journ.* Nov. 1. |
| Mr. Clarke—On the Kundak Oil | *Ibid.* Nov. 1. |
| Dr. Griffith—On Pus and Mucus | *Med. Gaz.* Nov. 18. |

---

N.B. The selections from the British and Foreign Journals will be found less numerous than usual. It is intended hereafter to replace these partial extracts by a digested Report of the progress of each department of medical science during the preceding twelve months. The first of these Reports will appear in the October Number.

